# Supplementary material for: A Pilot Feasibility Study Assessing the Combined Effects of Early Behavioral Intervention and Propranolol on Autism Spectrum Disorder (ASD)
Source: Children (Basel). 2023 Sep 30;10(10):1639. doi: 10.3390/children10101639 (PMC10605265; doi:10.3390/children10101639)
Supplement: Supplementary file 1 [file children-10-01639-s001.zip › children-2597855-supplementary.pdf]

Supplementary Table S1. Summary of outcomes for all participants entering the study with last observations carried forward

Task	Propranolol-baseline	Propranolol-6wk	Propranolol-12wk	Placebo-baseline	Placebo-6wk	Placebo-12wk
GSOM total	32.0 (±15.3 SD)	27.6 (±9.7 SD)	41.2 (±14.0 SD)	19.0 (±4.1 SD)	16.0 (±7.2 SD)	18.8 (±9.1 SD)
SRS	74.8 (±11.2 SD)	79.4 (±19.9 SD)	78.2 (±17.0 SD)	78.8 (±9.6 SD)	76.8 (±10.9 SD)	77.3 (±10.6 SD)
PAS	76.3 (±20.1 SD)	80.0 (±22.9 SD)[1]	84.8 (±32.9 SD)	57.0 (±14.0 SD)	60.5 (±21.0 SD)	60.8 (±21.5 SD)
SEQ	90.8 (±23.9 SD)	102.6 (±25.5 SD)[2]	101.6 (±30.7 SD)[3]	107.3 (±21.4 SD)	101.3 (±13.5 SD)	100.3 (±21.9 SD)
Vineland-communication	78.8 (±20.3 SD)	73.2 (±16.3 SD)	73.6 (±26.2 SD)	67.0 (±16.5 SD)	68.3 (±17.1 SD)	66.8 (±19.4 SD)
Vineland-daily living	78.4 (±19.3 SD)	72.0 (±8.0 SD)	65.2 (±20.4 SD)	72.0 (±10.1 SD)	72.5 (±9.7 SD)	72.3 (±14.2 SD)
Vineland-social	70.4 (±13.8 SD)	67.0 (±5.7 SD)	68.8 (±10.2 SD)	60.0 (±17.5 SD)	60.5 (±15.6 SD)	60.3 (±15 8 SD)
AIM-Peer Interaction Subdomain Impact raw score	27.6 (±6.4 SD)	25.8 (±8.4 SD)	22.8 (±8.7 SD)[4]	27.8 (±6.0 SD)	27.3 (±7.6 SD)	26.8 (±5.1 SD)
AIM-Social Reciprocity Subdomain Impact raw score	30.4 (±1.1 SD)	31.2 (±4.7 SD)	30.4 (±3.1 SD)	37.0 (±9.4 SD)	34.0 (±11.9 SD)	34.3 (±10.0 SD)
AIM-Atypical Behavior Subdomain Impact raw score	32.8 (±15.0 SD)	35.2 (±8.4 SD)	37.6 (±15.3 SD)[5]	30.0 (6.5 SD)	30.8 (±6.7 SD)	29.5 (±6.2 SD)
AIM-Communication Subdomain Impact raw score	36.2 (±8.0 SD)	34.4 (±6.4 SD)	35.8 (±11.8 SD)	43.5 (±5.4 SD)	40.3 (±9.0 SD)	41.5 (±5.2 SD)
AIM-Repetitive Behavior Subdomain Impact raw score	41.2 (±19.1 SD)	43.6 (±14.8 SD)[6]	43.4 (±19.7 SD)	45.5 (8.3 ± SD)	40.3 (±5.7 SD)	43.3 (±10.6 SD)

(Student's paired t-test:
1 = p=0.070 vs baseline, 2 = p=0.051 vs baseline, 3 = p=0.064 vs baseline, 4 = p=0.087 vs baseline, 5 = p=0.080 vs baseline, 6 = p=0.061 vs baseline)
